# Can Neutrophil-to-Lymphocyte or Platelet-to-Lymphocyte Ratio Be Used to Predict Postoperative Nausea and Vomiting in Breast Reduction?

**DOI:** 10.7759/cureus.7237

**Published:** 2020-03-10

**Authors:** Onur Karaca, Guvenc Dogan

**Affiliations:** 1 Anesthesiology and Reanimation, Aksaray University, Aksaray, TUR; 2 Anesthesiology, Hitit University, Erol Olcok Training and Research Hospital, Corum, TUR

**Keywords:** breast reduction, neutrophil, lymphocyte, platelet, nausea, vomiting

## Abstract

Background/Objective

Postoperative nausea and vomiting (PONV) is one of the most frequently seen complications in the postoperative period. In several studies, the neutrophil-lymphocyte ratio (NLR) or the thrombocyte-lymphocyte ratio (PLR) has been suggested as a parameter to be used in the diagnosis of inflammatory diseases. However, the literature provides no information about this relationship for breast reduction. This study aims to investigate whether preoperative NLR or PLR was an indicator of PONV and identify its relationship with antiemetic use.

Methods

In this study, hemogram values and antiemetic amounts taken within 24 hours were obtained retrospectively by scanning the files of the patients received breast reduction diagnosis and operation. The confounder effect was controlled using the Propensity Score Matching analysis to distribute the case-control groups similarly. The Receiver Operating Characteristic (ROC) analysis was used to determine whether NLR and PLR could be a prognostic indicator for PONV prediction. Sensitivity and specificity values were calculated after the ROC analysis to determine the success of the cut-off points.

Results

The success of NLR and PLR in discriminating PONV was found to be statistically significant (cut-off: 1.97, area under the curve (AUC)=0.697, p=0.001, cut-off: 137.2, AUC=0.743; p<0.001, respectively). In addition, the sensitivity of PLR (77.8%) in discriminating PONV was found to be higher in comparison to NLR (73.3%).

Conclusions

One of the factors decreasing patient care quality and satisfaction is PONV. The results of this study showed that preoperative NLR and PLR could be taken into consideration in antiemetic use required for the prevention of postoperative nausea-vomiting in breast-reduction operations.

## Introduction

Postoperative nausea and vomiting (PONV) is defined as gagging or nausea-vomiting within the postoperative 24 hours [1-4].

The prevalence of nausea and vomiting changes according to the surgical cases performed under general anesthesia and, generally, its incidence is reported to be between 30% and 80% [2-4]. Nausea and vomiting are among the important problems that commonly occur in the postoperative period and that decreases patient satisfaction [5]. Anesthesia medicine developed in recent years decreases these effects significantly; in fact, some of the anesthesia medicine given intravenously before extubation aims to prevent this problem [6].

Despite the developments in the management of nausea and vomiting and new antiemetic medicine, postoperative nausea and vomiting remain to be an important problem for patients [2,4]. Several risk factors play an important role in PONV development [7-8]. PONV-related important risk factors include patient-related, anesthetic, and surgical factors. The most important patient-related risk factor is being female; and the other factors include having a history of nausea and vomiting in the postoperative period, not smoking, motion sickness history, and young age. Anesthetic factors that play a role in PONV development include the use of inhalation anesthesia (volatile), duration of anesthesia, use of postoperative opioids, liquid anesthetics (isoflurane, desflurane, sevoflurane), and gas anesthetics (nitric oxide (N_2_O), nitrous oxide) [2,9]. The type of surgery is reported to be an important risk factor in PONV development. PONV incidence is reported to be higher in some operation types (abdominal surgeries) depending on long-time exposure to anesthesia and the use of high dose opioids [2,10-11].

There are various factors related to the risk of nausea and vomiting, and inflammation increases this risk. In several studies, the neutrophil-lymphocyte ratio (NLR) or the thrombocyte-lymphocyte ratio (PLR) has been suggested as a parameter to be used in the diagnosis and follow-up of the inflammatory diseases [12-15]. However, although this relationship has been reported in only a limited number of studies for rhinoplasty in the literature, the literature provides no information about this relationship for breast reduction operations. This study aims to investigate whether preoperative NLR or PLR was an indicator of PONV and identify its relationship with antiemetic use.

## Materials and methods

In this study, hemogram values and antiemetic amounts (metoclopramide) taken within 24 hours postoperatively were obtained retrospectively by scanning the files of the patients who applied to the Plastic, Reconstructive and Aesthetic Surgery polyclinic and were scheduled for a breast reduction operation. Patients aged between 18 and 65 years, who underwent elective mammoplasty in the I-II risk group according to the American Society of Anesthesiologists (ASA) were involved in the study. None of the patients received a preoperative blood transfusion, and the patients had no history of gastrointestinal system disorders, uncontrolled systemic diseases, or antiemetic and anticholinergic medicine use. Patients who were treated with pre/perioperative steroids and antiemetics and who underwent intraoperative local anesthesia were excluded from the study. All patients’ oral intake was restricted eight hours before the operation. All the operations were performed by the same surgeon.

Electrocardiography, peripheral oxygen saturation, noninvasive blood pressure, end-tidal carbon dioxide pressure, and body temperature monitoring were conducted in all patients. The anesthesia induction of all patients was conducted with 2-3 mg kg-1 propofol, 0.6-0.8 mg kg-1 rocuronium bromide, and 1 µg kg-1 remifentanil. In the maintenance of anesthesia, 2%-2.5% sevoflurane was given with a 50% air/O_2_ mixture, and 0.05-0.2 µg kg-1 min-1 remifentanil infusion and rocuronium bromide as a muscle relaxant as needed were used. After the surgical procedure, for postoperative analgesia, 1 g metamizole sodium was used and all patients in whom sugammadex was used to reverse neuromuscular blockade, and the patients were transferred to the post-anesthesia care unit (PACU) after extubation.

Ethics committee approval dated 2019 and numbered 115 was obtained from the Ethics Committee of Hitit University, and the study followed the Declaration of Helsinki.

Sample size estimations (Priori power analysis) and power analysis

The sample size was calculated using G-power (Version 3.1; (Heinrich-Heine-Universität Düsseldorf, Düsseldorf, Germany)) package programming. The sample size was calculated for the student's t-test, which was used for testing the main hypothesis of the present study. It was found that 72 individuals, 36 in two different groups, needed to be involved in the study in order to reveal the significant differences in the groups (NLR ≥ 2 and NLR < 2) using 80% power (1-β=0.80), α=0.05 error (95% confidence interval), and 0.6 effect size with a one-sided hypothesis.

Statistical analysis and control of confounding effect in study design

As all the patients in the groups were female, gender did not have a confounding role. The confounder effect was controlled using the Propensity Score Matching (PSM) analysis in order to distribute the case-control groups similarly in terms of statistical comparisons. Prior to the study, the confounder between the case-control groups was matched in terms of the continuous variables, age, body mass index (BMI), smoking, and additional diseases. Propensity scores were predicted using the combined estimator (ensemble learning: a combination of logistic regression and machine learning algorithms) developed by Demir E (2019) [16]. A matching analysis was performed using the Nearest Neighbor Matching method. The case-control ratio was matched equally as 1:1 in the matching analysis. After the matching analysis based on the propensity score, the control of the balance was evaluated using the overall chi-square balance test [17]. Before the matching, there were 75 patients in the NLR < 2 group and 46 patients in the NLR ≥ 2 group. After the matching, analyses were performed with a total of 92 patients' data, with 46 patients distributed to two different groups equally according to confounder variables. A matching analysis was performed in the R package with the "Matching" library, and graphic drawings were done with the “ggplot 2” library [18].

The statistical analysis was performed using the SPSS (Version 22.0, SPSS Inc., Chicago, IL, USA) package program. Descriptive statistics were reported as mean ± standard deviation (SD) or median (minimum-maximum) according to data normality distribution for continuous variables. Descriptive statistics of categorical data were presented as numbers and percentages. The normality distribution of the data was evaluated by the Kolmogorov-Smirnov and Shapiro-Wilk tests.

The patients that had NLR hemogram values below and above 2 based on the cut-off point suggested in the literature were divided into two groups; antiemetic use averages and nausea and vomiting ratios within 24 hours were compared statistically. Demographic characteristics and antiemetic use comparisons of the independent two groups (NLR ≥ 2 and NLR < 2) were performed using the student's t-test or nonparametric Mann Whitney U test according to data distribution; PONV ratio comparisons were done using the chi-square test. In addition, whether NLR and PLR could be a prognostic indicator for PONV was investigated using the receiver operating characteristic (ROC) curve. The area under the ROC curve (AUC) was evaluated as 0.9-1: Excellent, 0.8-0.9: Good, 0.7-0.8: Fair, 0.6-0.7: Poor, and 0.5-0.6: Fail. After the ROC analysis, the Youden index (maximum sensitivity and specificity) was used in order to identify the best cut-off point. When it was significant, sensitivity, specificity, positive-negative predictive values, and likelihood ratio (+) values were calculated using the cut-off points after the ROC analysis in order to identify the distinguishing power of the indicator. The statistical significance level was accepted as p < 0.05. 

## Results

All the patients in the study were female. After the matching, the groups were distributed similarly in terms of age, BMI, surgery duration, ratios of smoking, and ASA (p=0.243, p=0.405, p= 0.119, p=0.656, p=0.524, respectively; Table 1). Group 1 preoperative NLR average: 1.59±0.36 was significantly lower than Group 2 NLR average: 3.99±1.57 (p < 0.001).

**Table 1:** Comparison of demographic characteristics and clinic findings NLR: neutrophil-to-lymphocyte ratio, VAS: visual analog scale, ASA: The American Society of Anesthesiologists scale, BMI: body mass index a Student’s t-test, b Chi-square test

	n	Age (years)	BMI	Duration of surgery	Smoking (Yes/No)	ASA (I/II)	VAS
Group 1 (NLR<2)	46	42.03±8.93	30.17±4.38	1.96±0.37	14/32	20/26	4.54±1.81
Group 2 (NLR≥2)	46	39.97±7.84	29.45±3.86	2.09±0.42	16/30	17/29	4.87±1.89
P values		0.243^a^	0.405^a^	0.119^a^	0.656^b^	0.524^b^	0.401^a^

Antiemetic use in 24 h averages and nausea-vomiting ratios were distributed significantly different between the groups (p=0.009, p=0.002, Table 2). Antiemetic use of the group with NLR<2 was 8.00±9.27, which was 13.42±10.12 lower than the group average of the group with NLR≥2. In addition, 32 (69.6%) patients in the NLR < 2 group did not have nausea/vomiting, and 14 (30.4%) patients had nausea or vomiting. As for the group with NLR≥2, 15 (32.6%) patients did not have nausea/vomiting and 31 (67.4%) patients had nausea and vomiting.

**Table 2:** Comparison of antiemetic use and postoperative nausea-vomiting according to NLR (neutrophil-to-lymphocyte ratio) groups a: Mann Whitney U test, b: Chi-square test

	n	Antiemetic use in 24 h (mg)	P-value	Postoperative nausea-vomiting			P-value
				No	Only Nausea	Vomiting	
Group 1 (NLR<2)	46	8.00±9.27	0.009^a^	32 (69.6%)	10 (21.7%)	4 (8.7%)	0.002^b^
Group 2 (NLR≥2)	46	13.42±10.12		15 (32.6%)	21 (45.7%)	10 (21.7%)	

**Figure 1:**
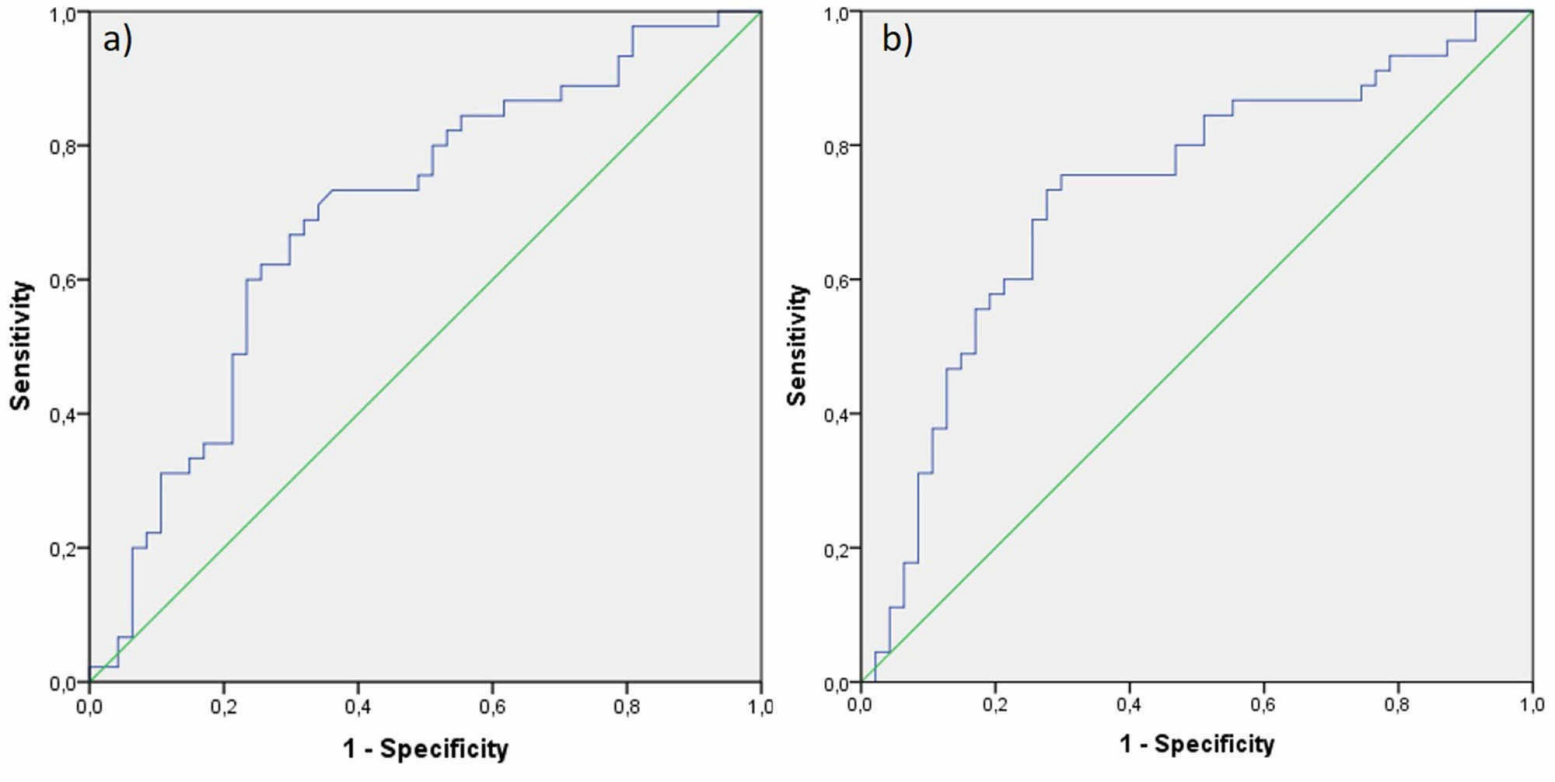
ROC curves obtained for the predicting success of NLR and PLR parameters in a PONV (postoperative nausea and vomiting) diagnosis (a) ROC curves of neutrophil-to-lymphocyte (b) platelet-to-lymphocyte ratio ROC: receiver operating characteristic; PONV: postoperative nausea and vomiting

**Figure 2:**
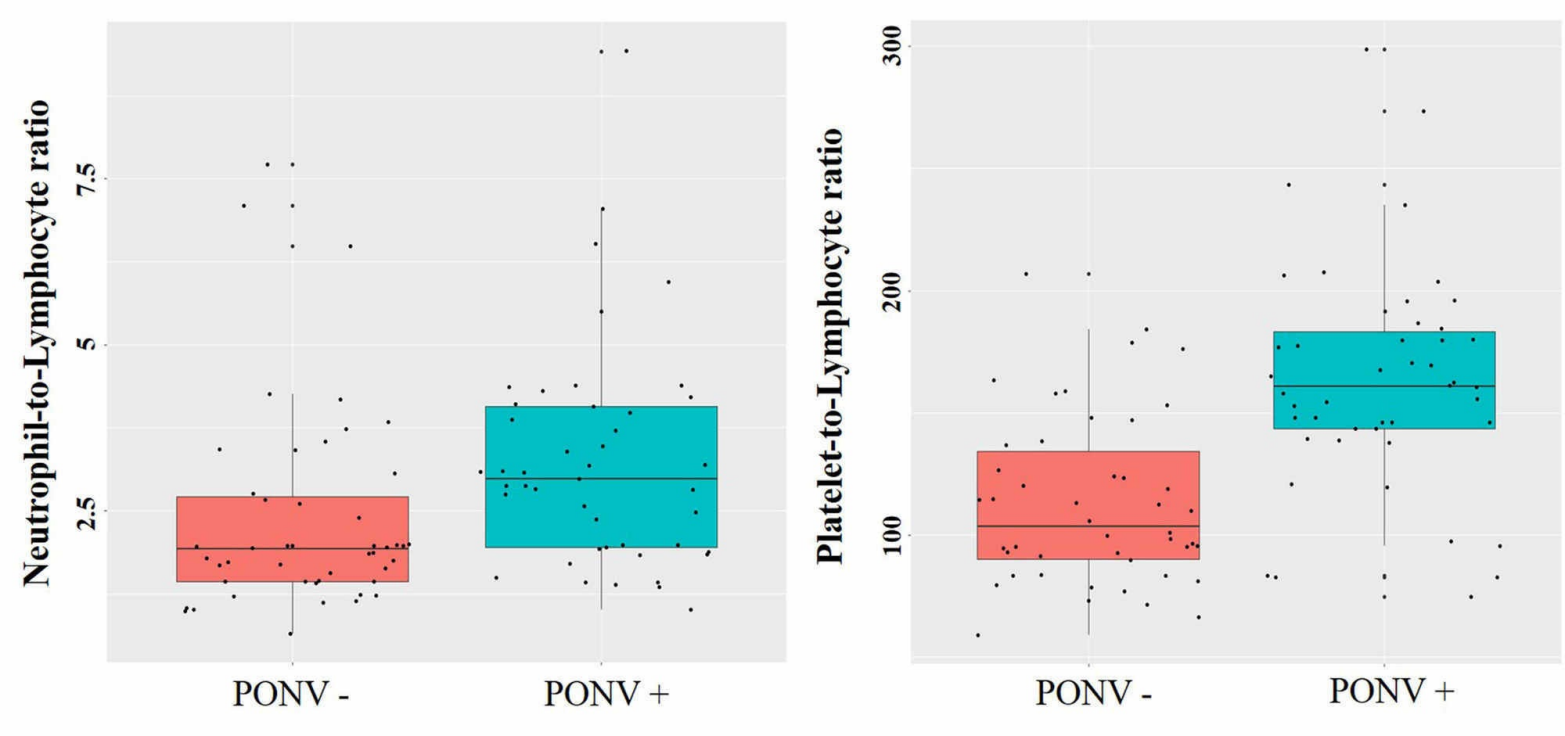
Box plots of neutrophil-to-lymphocyte and platelet-to-lymphocyte ratio according to the postoperative nausea and vomiting groups (a) Box plot of neutrophil-to-lymphocyte (b) platelet-to-lymphocyte ratio

Figure 1 shows the ROC curves obtained for the predicting success of NLR and PLR parameters in PONV diagnosis while Figure 2 shows the box plots. Table 3 shows the sensitivity, specificity, positive-negative predictive values and likelihood ratio (+) values identified after the ROC analysis. The area under ROC curve was significant for NLR in discrimination (AUC=0.697 (0.588-0.805); p=0.001). When NLR was higher than 1.97, its sensitivity in the PONV diagnosis was found to be 0.733 (0.578-0.849) and its specificity was found to be 0.638 (0.485-0.769). The area under the ROC curve was significant for PLR in the discrimination (AUC=0.743 (0.639-0.847); p<0.001). When PLR was higher than 137.2, its sensitivity in the PONV diagnosis was found to be 0.778 (0.625-0.883), and its specificity was found to be 0.702 (0.549-0.822). The other values are given in Table 3. According to the area under the ROC curve values, NLR and PLT predicted “good level” PONV.

**Table 3:** ROC curve results and sensitivity, specificity, positive-negative predictive, and likelihood ratio (+) values NLR: neutrophil-to-lymphocyte ratio, PLR: platelet-to-lymphocyte ratio, AUC: area under curve, PPV: positive predictive value, NPV: negative predictive value, LR: likelihood ratio

	NLR	PLR
AUC (95 % CI)	0.697 (0.588-0.805)	0.743 (0.639-0.847)
P-values	0.001	<0.001
Cut off	≥ 1.97	≥ 137.2
Sensitivity	0.733 (0.578-0.849)	0.778 (0.625-0.883)
Specificity	0.638 (0.485-0.769)	0.702 (0.549-0.822)
PPV	0.660 (0.511-0.784)	0.714 (0.565-0.830)
NPV	0.714 (0.552-0.838)	0.767 (0.610-0.877)
LR +	2.03 (1.33-3.08)	2.61 (1.64-4.16)

## Discussion

In their retrospective study with 433 patients who had hyperemesis gravidarum and a control group of 160, Tayfur et al. (2017) investigated the relationship between the platelet-to-lymphocyte ratio, plateletcrit, and hyperemesis gravidarum (HG). The results of the study showed that the platelet-to-lymphocyte ratio and plateletcrit were effective inflammatory indicators in predicting HG presence, and they stated that thrombocyte levels could be used in identifying HG severity [19].

Arpacı et al. (2017) investigated the relationship between NLR and PONV in patients who underwent maxillofacial surgery and reported that PONV risk increased significantly in patients with a higher NLR. By stating that NLR could easily be calculated with data obtained from a full blood count and might be an indicator for PONV, they claimed that antiemetic prophylaxis could be given after the evaluation of the NLR ratio [20].

In their study conducted with 80 patients, Altun et al. (2019) grouped the patients retrospectively according to their NLR values below and above 2. They compared nausea vomiting at recovery, antiemetic requirement at recovery, and at 24 h postoperatively between the groups and reported statistically significant differences. The results showed that NLR values over 2 calculated in the preoperative period could be an indicator of PONV risk, and they claimed that antiemetic prophylaxis could be given according to this value [3]. The present study indicates similar results; it was found that antiemetic use in 24 h and nausea/vomiting ratios were significantly higher in the group with higher NLR values. However, in addition to the study conducted by Altun et al., the present study also investigated a new cut-off point with the ROC curve, and calculated sensitivity, specificity, PPV, and NPV values for the success of NLR. The success of NLR in discriminating PONV for the 1.97 cut-off point was found to be statistically significant. The cut-off point recommended as 2 for NLR in literature is in line with the 1.97 cut-off point we obtained as a result of the ROC curve.

The superiority of the present study among the ones in literature is that it shows that PLR could be used as a prognostic indicator in nausea/vomiting. A limited number of studies in the literature has investigated NLR, but no studies have been found to have the success of the PONV prediction of PLR. As for this study, a new cut-off point for PLR has been investigated and the success of PLR in discriminating PONV for the 137.2 cut-off point was found to be statistically significant. In addition, the sensitivity of PLR (77.8%) in discriminating PONV for this cut-off point was found to be higher in comparison to NLR (73.3%). The comparison of area values of NLR and PLR under the ROC curve showed that the success of PLR in predicting PONV was higher than NLR. According to ROC AUCs, NLR predicted fair and PLT predicted good PONV.

The limitation of the present study is that it was designed retrospectively as case-control. However, a matching analysis based on propensity score has been widely used in the literature recently. The analysis enables the similar distribution of the confounders, which decreases the bias in retrospective studies. The success of particularly PLR in predicting PONV could be investigated in new prospective studies to be designed.

## Conclusions

In our study, we showed that NLR and PLR can be predictors of PONV estimation. Based on our results, we have demonstrated that PLR is more sensitive and specific in predicting this complication. We think that these hemogram parameters should be taken into account in order to prevent this important and difficult to predict complication and to take necessary measures.
